# Supplementary material for: Diverse modes of H3K36me3-guided nucleosomal deacetylation by Rpd3S
Source: Nature. 2023 Jul 19;620(7974):669–75. doi: 10.1038/s41586-023-06349-1 (PMC10432269; doi:10.1038/s41586-023-06349-1)
Supplement: Supplementary file 1 — Raw data for all figures. [file 41586_2023_6349_MOESM1_ESM.pdf]

nature portfolio

https://doi.org/10.1038/s41586-023-06349-1

Supplementary information

Diverse modes of H3K36me3-guided nucleosomal deacetylation by Rpd3S

In the format provided by the authors and unedited

Supplementary Figure 1

Raw data for Figure 5

Figure 5a

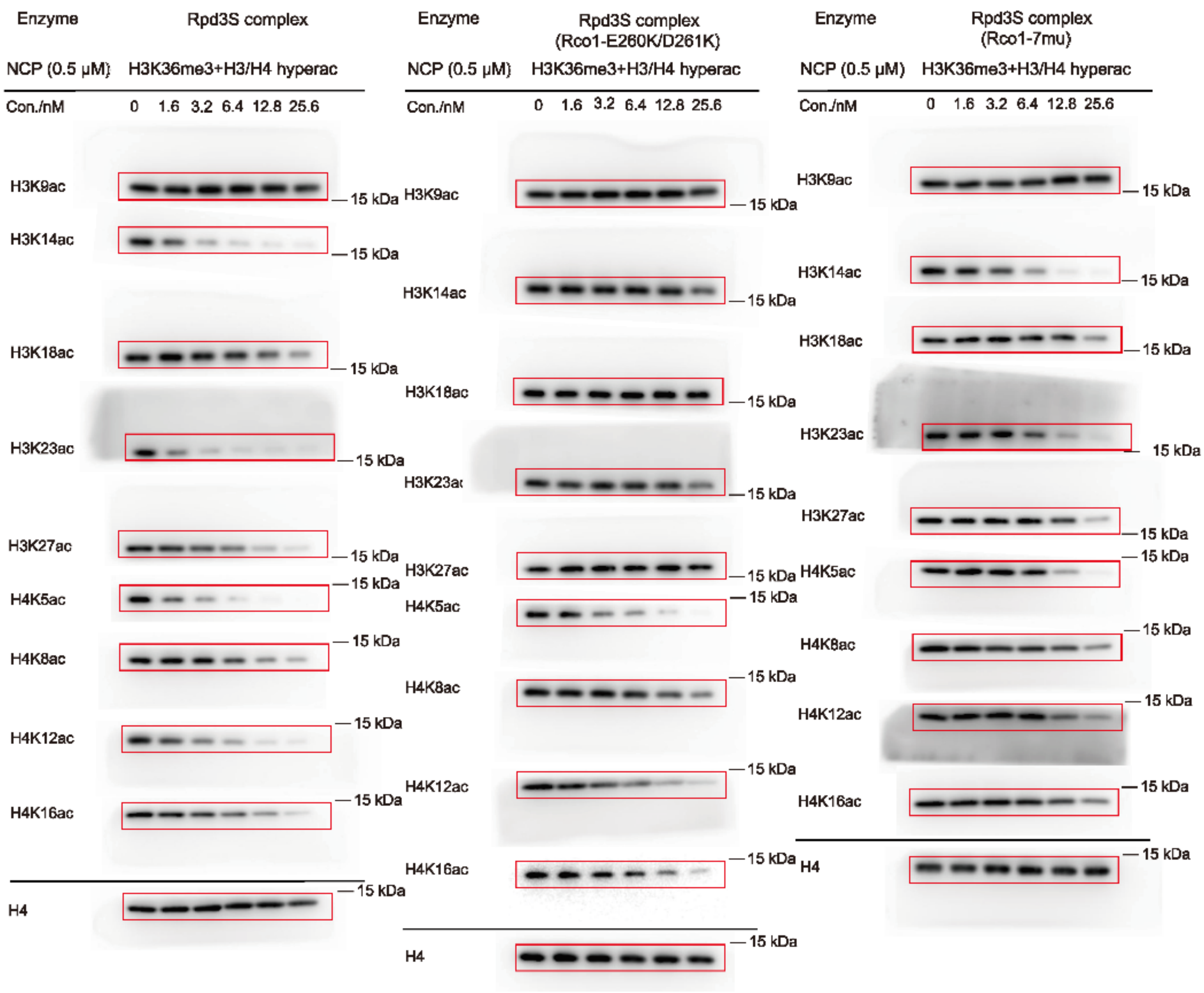

Raw data for Figure 6

Figure 6a

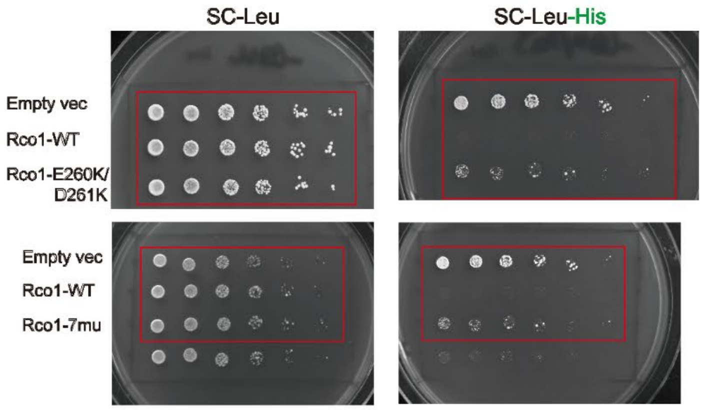

Figure 6b

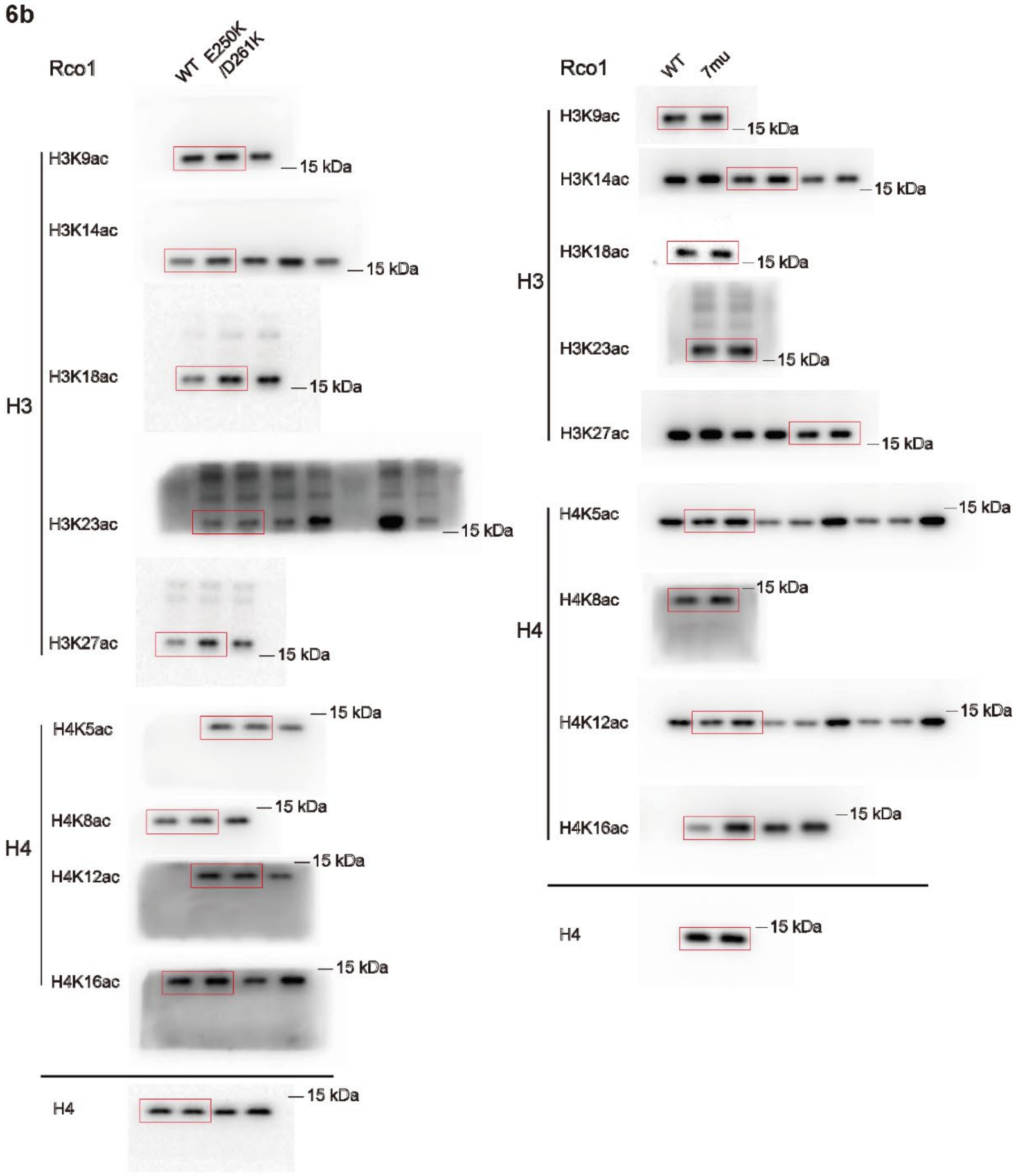

Raw data for Figure 6

Figure 6c

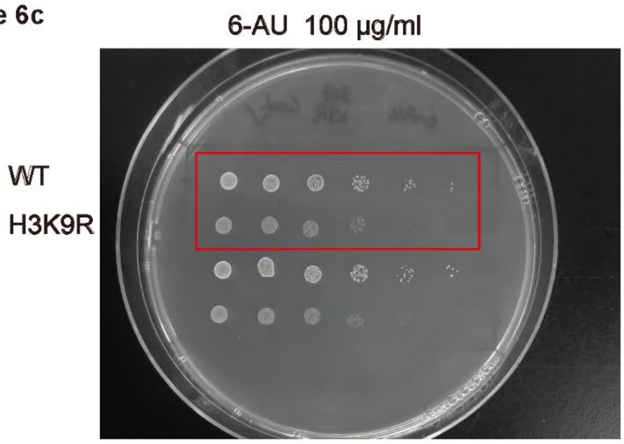

Figure 6d

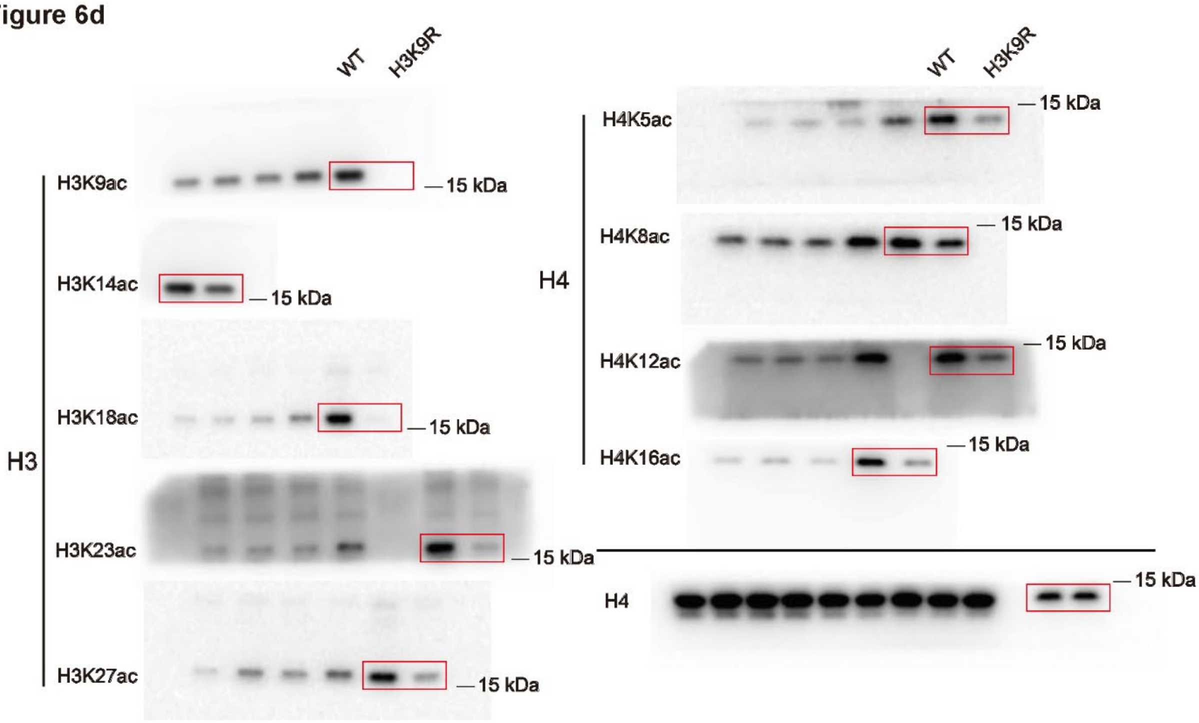

Raw data for Extended Data Figure 1

Extended Data Figure 1a

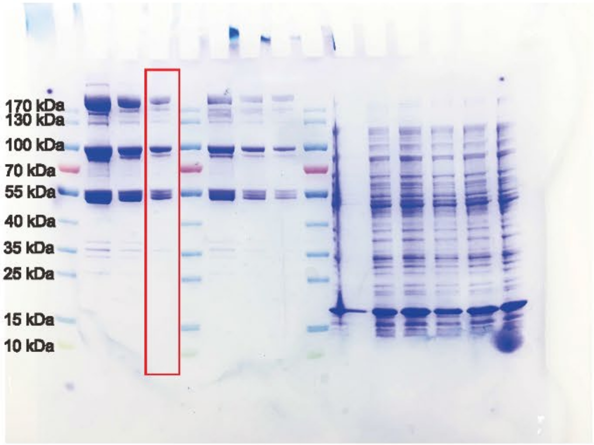

Extended Data Figure 1b

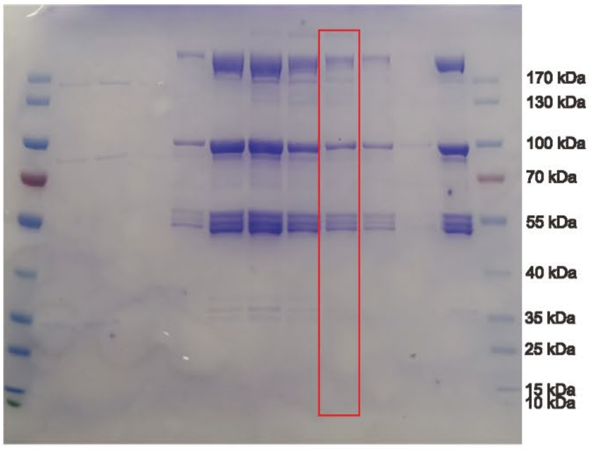

Extended Data Figure 1c

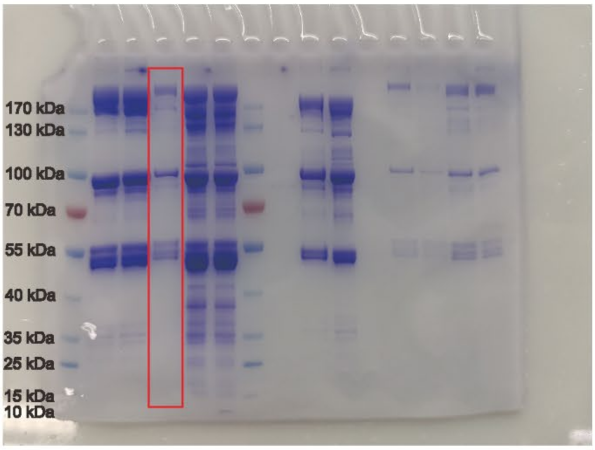

Raw data for Extended Data Figure 8

Extended Data Figure 8a

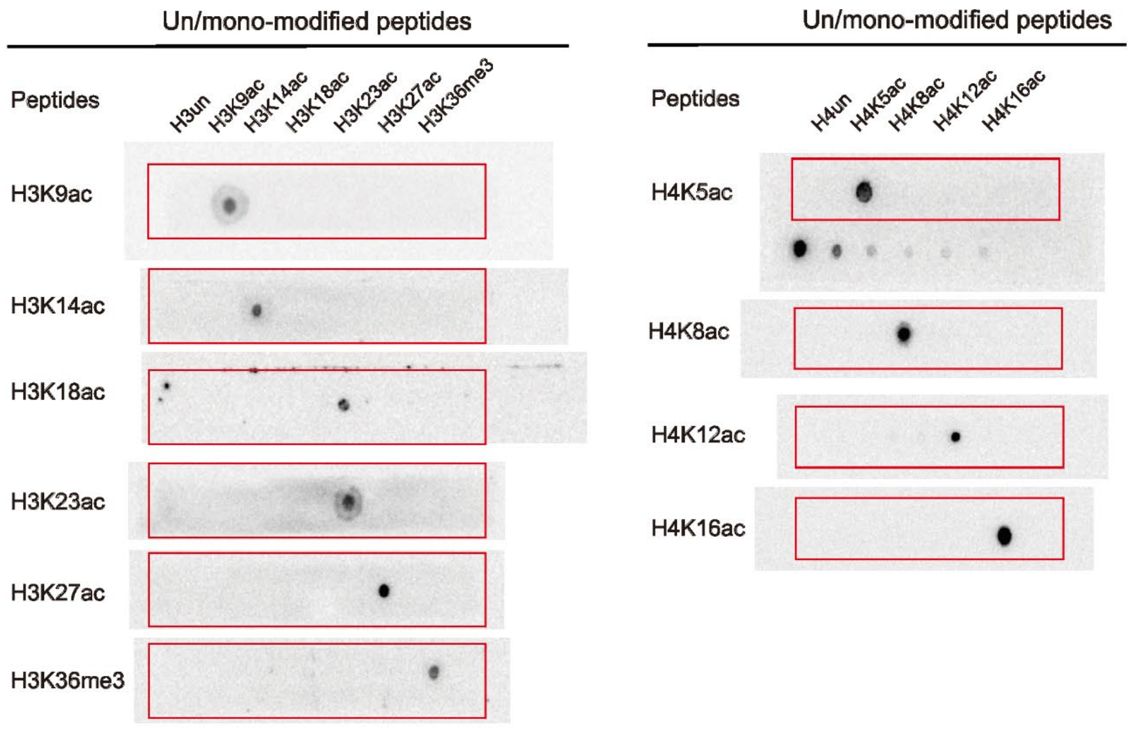

Raw data for Extended Data Figure 8

Extended Data Figure 8b

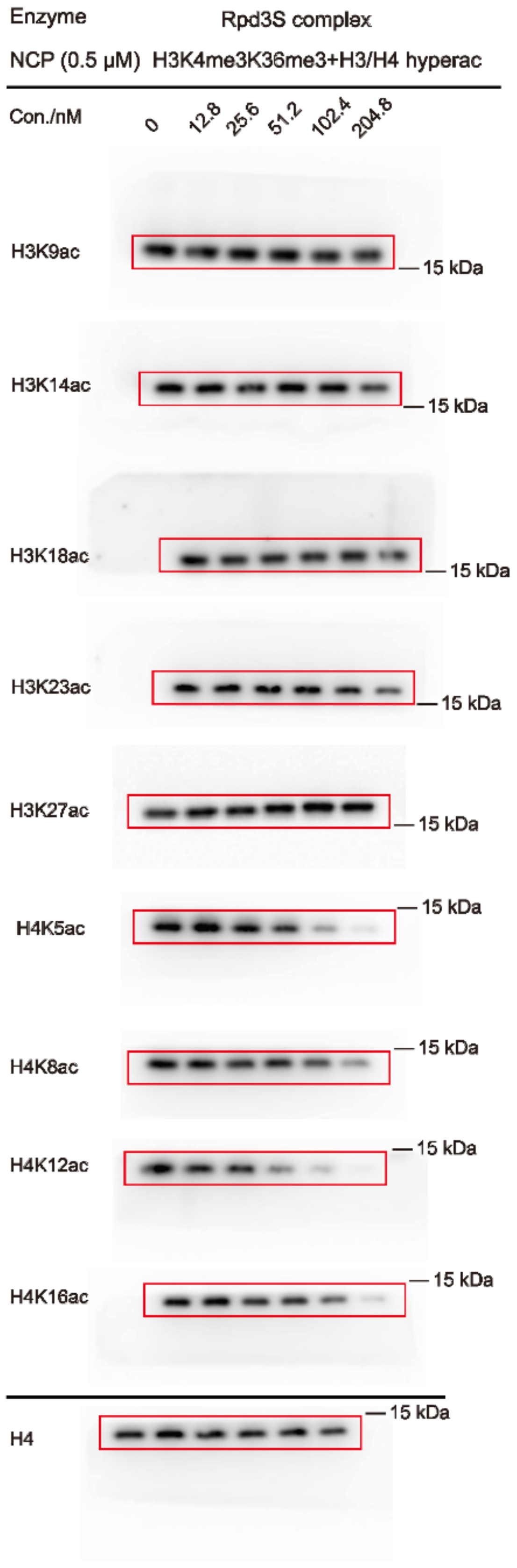

Extended Data Figure 8c

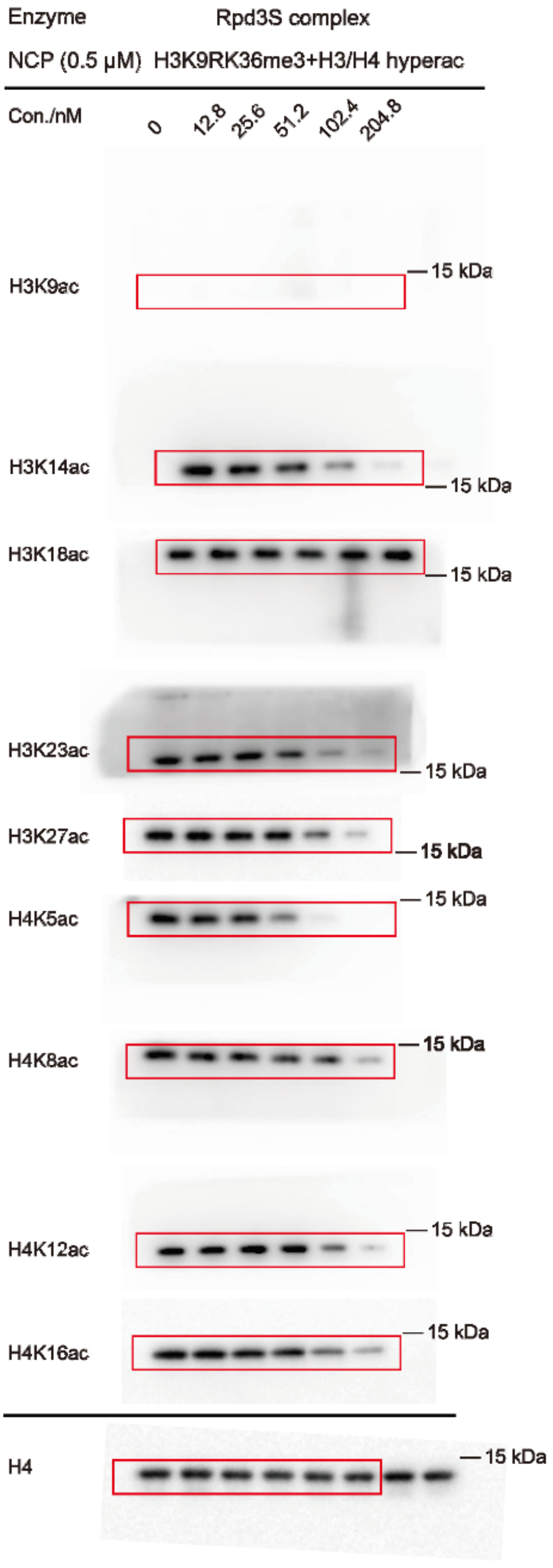

Raw data for Extended Data Figure 8

Extended Data Figure 8d

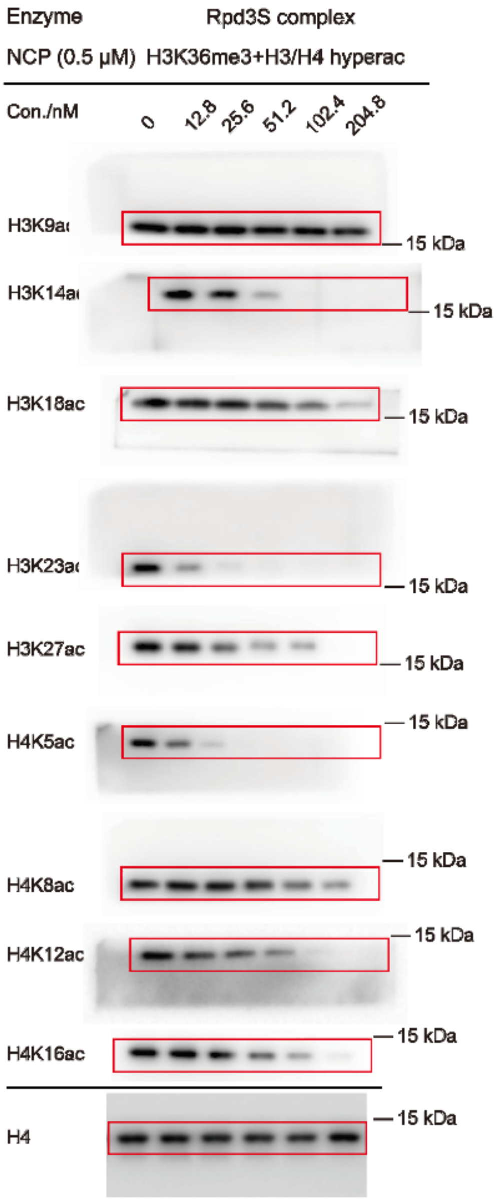

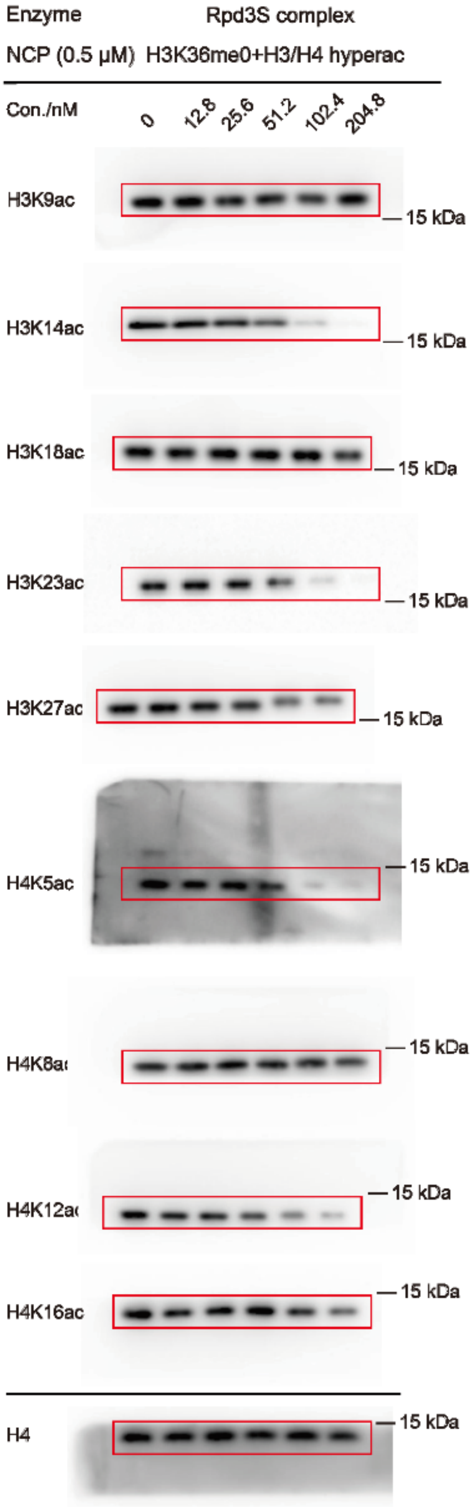

Raw data for Extended Data Figure 8

Extended Data Figure 8e

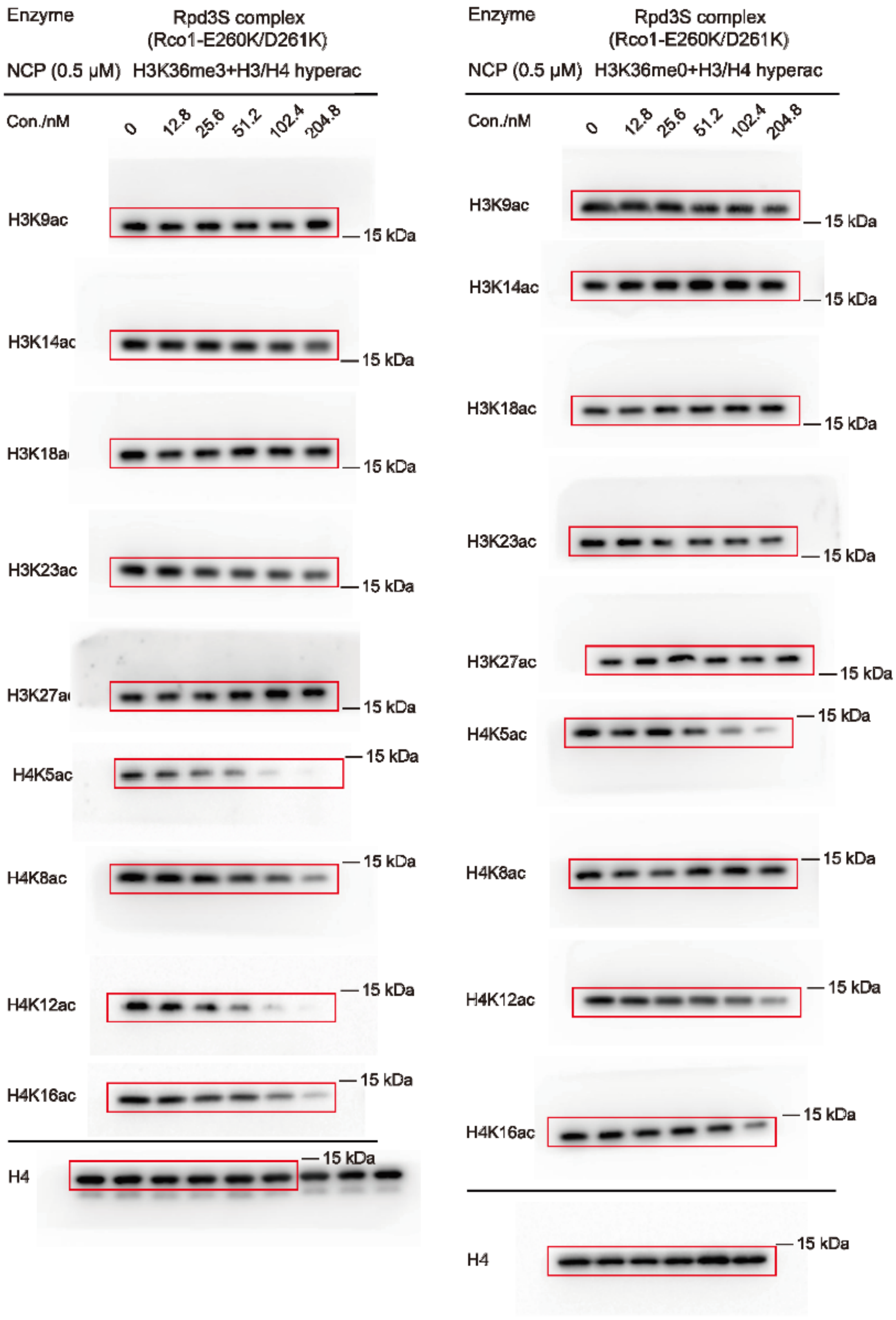

Raw data for Extended Data Figure 8

Extended Data Figure 8f

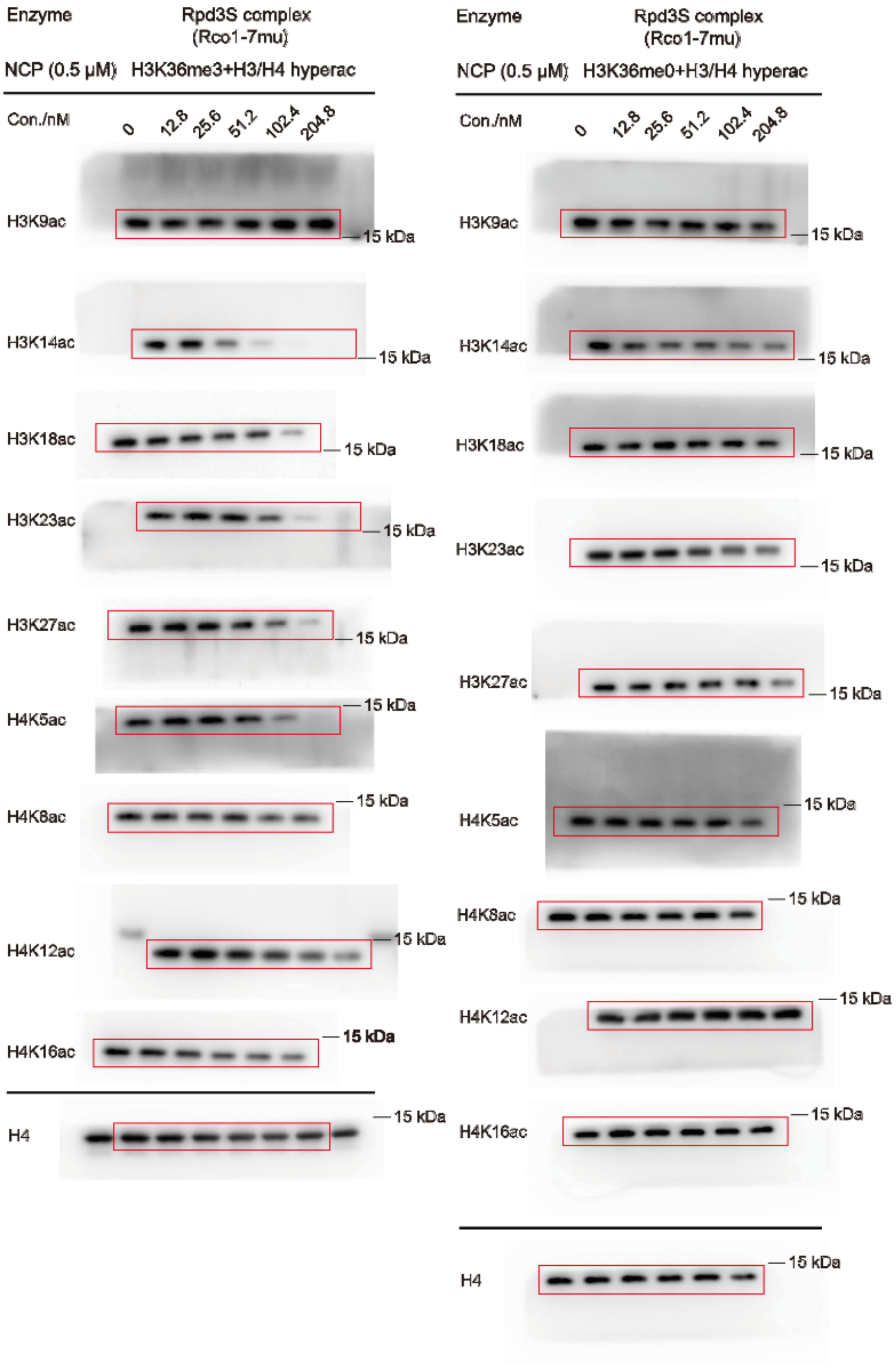

Raw data for Extended Data Figure 9

Extended Data Figure 9a

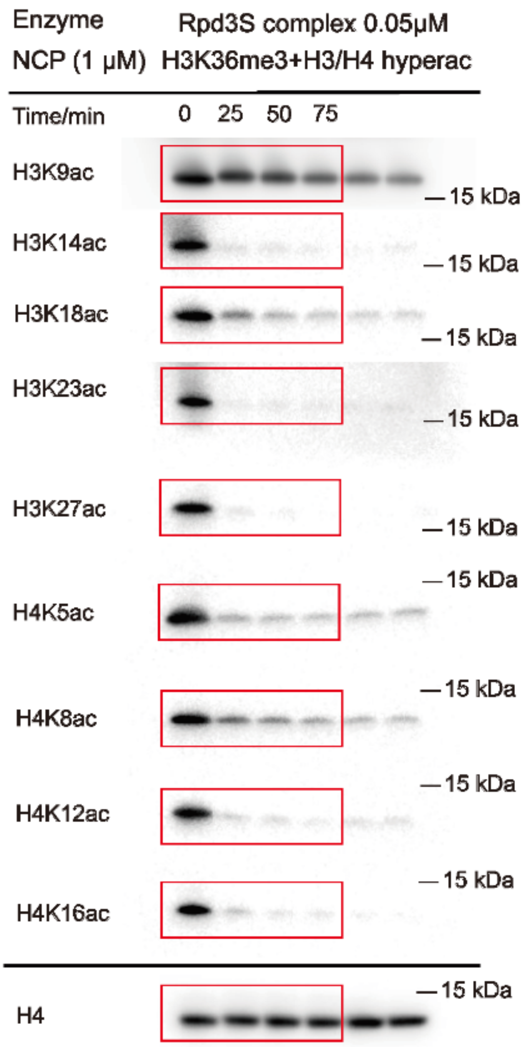

Extended Data Figure 9c

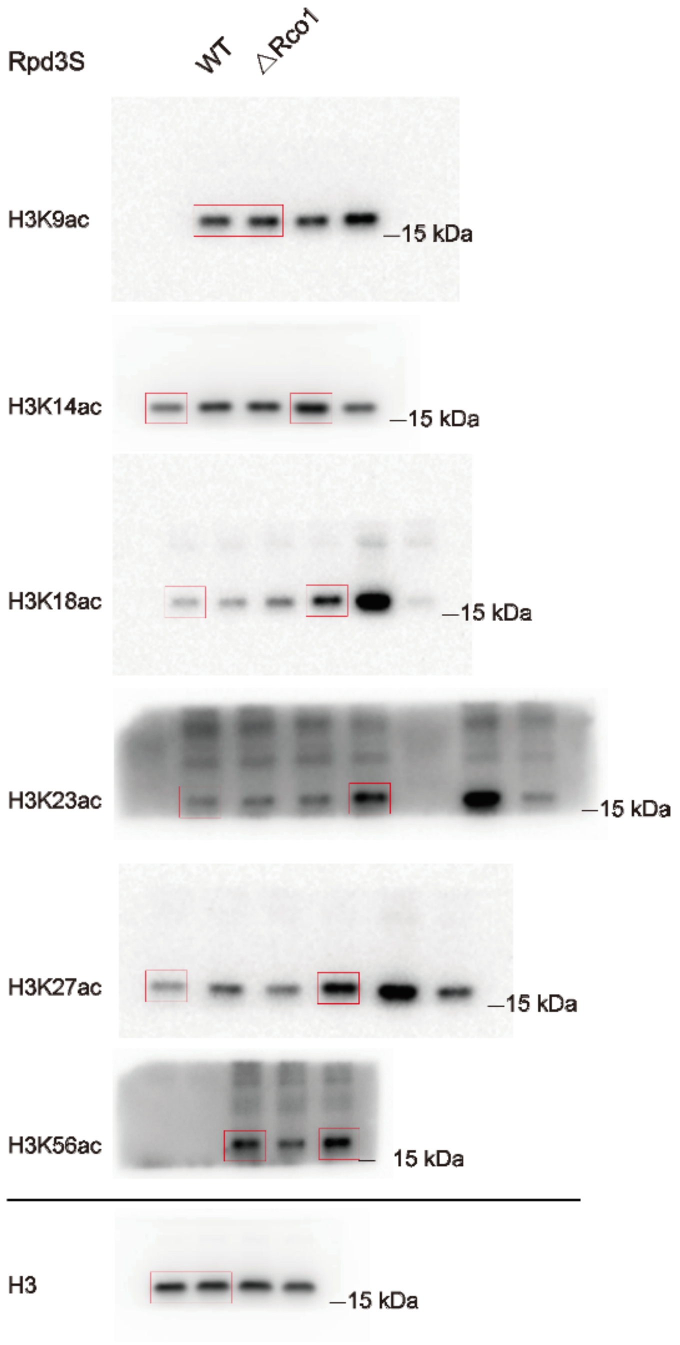

Supplementary Table 1. The yeast strains used in this study

Strains	Genotypes	Source
W303-1a	*MATa, leu2-3, 112, ura3-1, his3-11,15, trp1-1, ade2-1, can1-100*	Q. Li
YBL534	*MATa,his3Δ1 leu2Δ0 met15Δ0 ura3Δ0* ***RCO1Δ::KANMX6***	B. Li
YBL853	*MATa, his3Δ1 leu2Δ0 met15Δ0 ura3Δ0* ***RCO1Δ::KANMX6 STE11-1870::HIS3***	B. Li
