# Supplementary material for: Diverse modes of H3K36me3-guided nucleosomal deacetylation by Rpd3S
Source: Nature. 2023 Jul 19;620(7974):669–75. doi: 10.1038/s41586-023-06349-1 (PMC10432269; doi:10.1038/s41586-023-06349-1)
Supplement: Supplementary file 2 — Reporting Summary [file 41586_2023_6349_MOESM2_ESM.pdf]

nature portfolio

Corresponding author(s): Haitao Li

Last updated by author(s): May 31, 2023

Reporting Summary

Nature Portfolio wishes to improve the reproducibility of the work that we publish. This form provides structure for consistency and transparency in reporting. For further information on Nature Portfolio policies, see our Editorial Policies and the Editorial Policy Checklist.

Statistics

For all statistical analyses, confirm that the following items are present in the figure legend, table legend, main text, or Methods section.

n/a	Confirmed	
☐	☒	The exact sample size (*n*) for each experimental group/condition, given as a discrete number and unit of measurement
☐	☒	A statement on whether measurements were taken from distinct samples or whether the same sample was measured repeatedly
☒	☐	The statistical test(s) used AND whether they are one- or two-sided<br>*Only common tests should be described solely by name; describe more complex techniques in the Methods section.*
☒	☐	A description of all covariates tested
☒	☐	A description of any assumptions or corrections, such as tests of normality and adjustment for multiple comparisons
☐	☒	A full description of the statistical parameters including central tendency (e.g. means) or other basic estimates (e.g. regression coefficient) AND variation (e.g. standard deviation) or associated estimates of uncertainty (e.g. confidence intervals)
☒	☐	For null hypothesis testing, the test statistic (e.g. *F*, *t*, *r*) with confidence intervals, effect sizes, degrees of freedom and *P* value noted<br>*Give P values as exact values whenever suitable.*
☒	☐	For Bayesian analysis, information on the choice of priors and Markov chain Monte Carlo settings
☒	☐	For hierarchical and complex designs, identification of the appropriate level for tests and full reporting of outcomes
☒	☐	Estimates of effect sizes (e.g. Cohen's *d*, Pearson's *r*), indicating how they were calculated

Our web collection on statistics for biologists contains articles on many of the points above.

Software and code

Policy information about availability of computer code

Data collection	We used AutoEMation (version2.0) to collect all cryo-EM datasets, written by Dr. Jianlin Lei at Tsinghua University.
Data analysis	MotionCor2, GCTF v1.06, RELION 3.1.3, UCSF Chimera 1.16, UCSF Chimera X 1.2.5, COOT 0.9, Phenix-1.15.2-3472, Pymol 2.3.2, MolProbity 4.2

For manuscripts utilizing custom algorithms or software that are central to the research but not yet described in published literature, software must be made available to editors and reviewers. We strongly encourage code deposition in a community repository (e.g. GitHub). See the Nature Portfolio guidelines for submitting code & software for further information.

Data

Policy information about availability of data

All manuscripts must include a data availability statement. This statement should provide the following information, where applicable:

- Accession codes, unique identifiers, or web links for publicly available datasets
- A description of any restrictions on data availability
- For clinical datasets or third party data, please ensure that the statement adheres to our policy

The authors declare that the data supporting the findings of this study are available within the paper. The cryo-EM density maps have been deposited in the Electron Microscopy Data Bank under EMD accession codes 33845, 33846, 33847, 33848, 33849, 33850, 33851, 33852. The coordinates of the atomic model have been deposited in the Protein Data Bank under ID codes 7YI0, 7YI1, 7YI2, 7YI3, 7YI4, 7YI5. Several structural coordinates in the PDB database were used in this study, which can be located by accession numbers 6ESF and 3E9G. Source Data is available online.

Research involving human participants, their data, or biological material

Policy information about studies with human participants or human data. See also policy information about sex, gender (identity/presentation), and sexual orientation and race, ethnicity and racism.

Reporting on sex and gender	*Use the terms sex (biological attribute) and gender (shaped by social and cultural circumstances) carefully in order to avoid confusing both terms. Indicate if findings apply to only one sex or gender; describe whether sex and gender were considered in study design; whether sex and/or gender was determined based on self-reporting or assigned and methods used. Provide in the source data disaggregated sex and gender data, where this information has been collected, and if consent has been obtained for sharing of individual-level data; provide overall numbers in this Reporting Summary. Please state if this information has not been collected. Report sex- and gender-based analyses where performed, justify reasons for lack of sex- and gender-based analysis.*
Reporting on race, ethnicity, or other socially relevant groupings	*Please specify the socially constructed or socially relevant categorization variable(s) used in your manuscript and explain why they were used. Please note that such variables should not be used as proxies for other socially constructed/relevant variables (for example, race/ethnicity should not be used as a proxy for socioeconomic status). Provide clear definitions of the relevant terms used, how they were provided (by the participants/respondents, the researchers, or third parties), and the method(s) used to classify people into the different categories (e.g. self-report, census or administrative data, social media data, etc.) Please provide details about how you controlled for confounding variables in your analyses.*
Population characteristics	*Describe the covariate-relevant population characteristics of the human research participants (e.g. age, genotypic information, past and current diagnosis and treatment categories). If you filled out the behavioural & social sciences study design questions and have nothing to add here, write "See above."*
Recruitment	*Describe how participants were recruited. Outline any potential self-selection bias or other biases that may be present and how these are likely to impact results.*
Ethics oversight	*Identify the organization(s) that approved the study protocol.*

Note that full information on the approval of the study protocol must also be provided in the manuscript.

Field-specific reporting

Please select the one below that is the best fit for your research. If you are not sure, read the appropriate sections before making your selection.

☒ Life sciences ☐ Behavioural & social sciences ☐ Ecological, evolutionary & environmental sciences

For a reference copy of the document with all sections, see nature.com/documents/nr-reporting-summary-flat.pdf

Life sciences study design

All studies must disclose on these points even when the disclosure is negative.

Sample size	Sample sizes were not pre-determined. Cryo-EM images were collected until structures of satisfactory quality were solved, which suggested sufficient sample size. For biochemical assays, we performed two to three replicates since the replications are successful.
Data exclusions	No data were excluded during structural analysis.
Replication	Our HDAC assays, spotting assays and western blotting were performed in two to three independent replicates, all attempts at replication were successful. No data was excluded.
Randomization	No group allocation was performed in structural experiments.
Blinding	Blinding was not performed as subjective analysis was not needed and no group allocation was performed for structural experiments.

Reporting for specific materials, systems and methods

We require information from authors about some types of materials, experimental systems and methods used in many studies. Here, indicate whether each material, system or method listed is relevant to your study. If you are not sure if a list item applies to your research, read the appropriate section before selecting a response.

Materials & experimental systems

n/a	Involved in the study
	☒ Antibodies
	☒ Eukaryotic cell lines
☒	Palaeontology and archaeology
☒	Animals and other organisms
☒	Clinical data
☒	Dual use research of concern
☒	Plants

Methods

n/a	Involved in the study
☒	ChIP-seq
☒	Flow cytometry
☒	MRI-based neuroimaging

Antibodies

Antibodies used

Anti-Acetyl-Histone H3 (Lys14) Mouse mAb (PTM BIO, Cat.#: PTM-157, Dilution: 1:500), Anti-Acetyl-Histone H3 (Lys18) Mouse mAb (PTM BIO, Cat.#: PTM- 158, Dilution: 1:750), Anti-Acetyl-Histone H3 (Lys23) Rabbit pAb(PTM BIO, Cat.#: PTM- 115, Dilution: 1:1500), Anti-Acetyl-Histone H3 (Lys27) Mouse mAb (PTM BIO, Cat.#: PTM- 160, Dilution: 1:2000),
Anti-Tri-Methyl-Histone H3 (Lys36) Rabbit pAb (PTM BIO, Cat.#: PTM- 625, Dilution: 1:1000), Anti-Histone H4 Mouse mAb (NT) (PTM BIO, Cat.#: PTM- 1009, Dilution: 1:1000), Histone H3 Rabbit pAb(ABclonal, Cat.#: A2348, Dilution: 1:2000), Anti-Acetyl-Histone H3 (Lys56) Mouse mAb (PTM BIO, Cat.#: PTM- 162, Dilution: 1:1000), Acetyl-Histone H3-K9 Rabbit pAb(ABclonal, Cat.#: A7255, Dilution: 1:5000), Acetyl-Histone H4-K8 Rabbit pAb (ABclonal, Cat.#: A7258, Dilution: 1:6000), Acetyl-Histone H4-K12 Rabbit mAb(ABclonal, Cat.#: A22754, Dilution: 1:1000), Anti-acetyl-Histone H4 (Lys5) Antibody, rabbit monoclonal(Sigma-Aldrich, Cat.#: 04-118, Dilution:1:5000), and Acetyl-Histone H4 (Lys16) (E2B8W) Rabbit mAb((Cell Signaling Technology, Cat.#: 13534S, Dilution:1:500).

Validation

The antibodies employed in this research were commercially obtainable and were authenticated by the provider based on the information presented in the relevant data sheets.
Anti-Acetyl-Histone H3 (Lys14) Mouse mAb (PTM BIO, Cat.#: PTM-157): https://www.ptmbiolabs.com/product/ptm-157/
Anti-Acetyl-Histone H3 (Lys18) Mouse mAb (PTM BIO, Cat.#: PTM- 158): https://www.ptmbiolabs.com/product/ptm-158/
Anti-Acetyl-Histone H3 (Lys23) Rabbit pAb (PTM BIO, Cat.#: PTM- 115): https://www.ptmbiolabs.com/product/ptm-115/
Anti-Acetyl-Histone H3 (Lys27) Mouse mAb (PTM BIO, Cat.#: PTM- 160): https://www.ptmbiolabs.com/product/ptm-160/
Anti-Tri-Methyl-Histone H3 (Lys36) Rabbit pAb (PTM BIO, Cat.#: PTM- 625): https://www.ptmbiolabs.com/product/ptm-625/
Anti-Histone H4 Mouse mAb (NT) (PTM BIO, Cat.#: PTM- 1009): https://www.ptmbiolabs.com/product/pan-h4-mouse-mab/
H3 Rabbit pAb(ABclonal, Cat.#: A2348): https://abclonal.com/catalog-antibodies/HistoneH3RabbitpAb/A2348
Anti-Acetyl-Histone H3 (Lys56) Mouse mAb (PTM BIO, Cat.#: PTM- 162): https://www.ptmbiolabs.com/product/ptm-162/
Acetyl-Histone H3-K9 Rabbit pAb(ABclonal, Cat.#: A7255): https://abclonal.com/catalog-antibodies/AcetylHistoneH3K9RabbitpAb/A7255
Acetyl-Histone H4-K8 Rabbit pAb (ABclonal, Cat.#: A7258): https://abclonal.com/catalog-antibodies/AcetylHistoneH4K8RabbitpAb/A7258
Acetyl-Histone H4-K12 Rabbit mAb(ABclonal, Cat.#: A22754): https://abclonal.com/catalog-antibodies/AcetylHistoneH4K12RabbitmAb/A22754
Anti-acetyl-Histone H4 (Lys5) Antibody, rabbit monoclonal(Sigma-Aldrich, Cat.#: 04-118): https://www.sigmaaldrich.cn/CN/en/product/mm/04118
Acetyl-Histone H4 (Lys16) (E2B8W) Rabbit mAb((Cell Signaling Technology, Cat.#: 13534S): https://www.cellsignal.com/products/primary-antibodies/acetyl-histone-h4-lys16-e2b8w-rabbit-mab/13534?_=1682335024811&Ntt=13534S&tahead=true

Eukaryotic cell lines

Policy information about cell lines and Sex and Gender in Research

Cell line source(s)	SF9, obtained from invitrogen
Authentication	Cell lines were directly purchased from Invitrogen. Cell line authentication was not performed at our hand during cell culture.
Mycoplasma contamination	Cell lines had been tested negative for mycoplasma contamination by Invitrogen before purchase. They were not tested at our hand during cell culture.
Commonly misidentified lines (See ICLAC register)	No commonly misidentified cell lines were used in the study.
